# Scalable spin Seebeck thermoelectric generation using Fe-oxide nanoparticle assembled film on flexible substrate

**DOI:** 10.1038/s41598-022-21200-9

**Published:** 2022-10-05

**Authors:** Yuichiro Kurokawa, Yusuke Tahara, Yuki Hamada, Masahiro Fujimoto, Hiromi Yuasa

**Affiliations:** 1 Graduate School and Faculty of Information Science and Electrical Engineering, Kyushu University, 744 Motooka, Nishi-ku, Fukuoka, 819-0395 Japan; 2 Graduate School of Science and Technology, Shinshu University, Ueda, Nagano 386-8567 Japan

**Keywords:** Spintronics, Magnetic devices

## Abstract

We fabricated Fe_3_O_4_ nanoparticle (NP)-assembled films on flexible polyimide sheets with Pt or Ta cap layer using a spin coating method and DC sputtering. The films were elaborated for spin Seebeck thermoelectric generator applications, and their spin Seebeck voltages were observed. We showed that the thermoelectric power of [Pt film/Fe_3_O_4_ NP]_*n*_ multilayered films increases with increasing number of stacking *n*. Additionally, we prepared spin Seebeck thermopile devices in which the Fe_3_O_4_ NP-assembled films capped by Pt and Ta are connected alternately in series. We demonstrated that spin Seebeck voltages of the thermopile devices are larger than those of single [Pt or Ta film/Fe_3_O_4_ NP]_*n*_ piece. Our results indicate that the spin Seebeck thermoelectric power of Fe_3_O_4_ NPs can be enhanced using a simple fabrication process without lithography technique.

Recently, spin-momentum-mediated heat-charge conversion technologies have been extensively studied for the development of thin thermoelectric generators and heat flow sensors^1–26^. Among these technologies, the thermoelectric generation (TEG) based on Spin Seebeck effect (SSE) is one of the most promising routes^1–5,7–13,15–20,22,24–26^. The SSE converts a temperature difference into a spin current in a magnetic material. When a heavy metal (HM) with large spin orbit coupling is attached to a magnetic material, the spin current generates an electric current through an inverse spin Hall effect (ISHE). In the case of conventional Seebeck effect (SE), the generated power is limited by electric and thermal conductivities following the Wiedemann–Franz law. This limitation is overcome using the SSETEG technology because the generated power is determined by thermal and magnon conductivities in magnetic material and electrical conductivity in HM, respectively. It means that the thermal conductivity and electrical conductivity can be controlled separately. Moreover, TEG devices can be made thinner when using SSE compared to conventional SE because the SSE voltage and temperature difference directions are orthogonal^13^. Thin TEG devices can achieve a sufficient flexibility allowing the use of heat sources with non-flat surfaces. To produce SSETEG with sufficient flexibility, plastic materials should be used as substrates for SSE devices. However, to obtain the fine crystalline structure, most thin magnetic films for the SSE are exposed to high temperatures either in annealing or during deposition processes (for example, *T* = 993–1033 K for Y_3_Fe_5_O_12_ (YIG)^4,15,24,25^, *T* = 1073 K for Bi doped YIG^9^, *T* = 723 K for Fe_3_O_4_^7,10,16,19,27^, *T* = 873–923 K for Gd_3_Fe_5_O_12_^17,26^, *T* = 873 K for NiFe_2_O_4_^8^, and *T* = 873 K for CoFe_2_O_4_^11^). In contrast, to realize flexible SSETEG devices, magnetic materials should be fabricated under near room temperature to avoid high-temperature-induced degradations of the plastic substrate (for example the polyimide is degenerated at *T* > 573 K)^28^. To solve the problem, ferrite plating method, which can create the ferrite film without annealing, has been proposed and SSE on the flexible sheet has been reported^12^.

In addition to the above method, magnetic nanoparticles (NPs) constitute excellent candidates for the fabrication of flexible SSETEG devices because these can be deposited on flexible plastic substrates at room temperature using spin coating methods. Additionally, the nano-sized crystalline material generally has low thermal conductivity^29^, which enhances thermoelectric performances. The thermoelectric power of conventional Seebeck devices is increased by low thermal conductivity originated from the small grain size^30^. In our previous study, we fabricated YIG NPs using the coprecipitation method and observed the SSE^31,32^. However, even in that case, additional annealing process at *T* > 1073 K was needed after spin coating to obtain crystalline YIG NPs^31,32^. Methods to obtain crystalline magnetic Fe_3_O_4_ NPs using organic solution-phase decomposition of the iron precursor have been reported^33^. In these methods, NPs are initially crystallized prior to spin coating, which eliminates the need for further high-temperature annealing. In this study, we focused on a process technology for flexible spin Seebeck device and fabricated Fe_3_O_4_ NP-assembled films on polyimide sheets using crystallized Fe_3_O_4_ NPs and observed the SSE voltage under a temperature difference.

## Experimental

**Figure 1:**
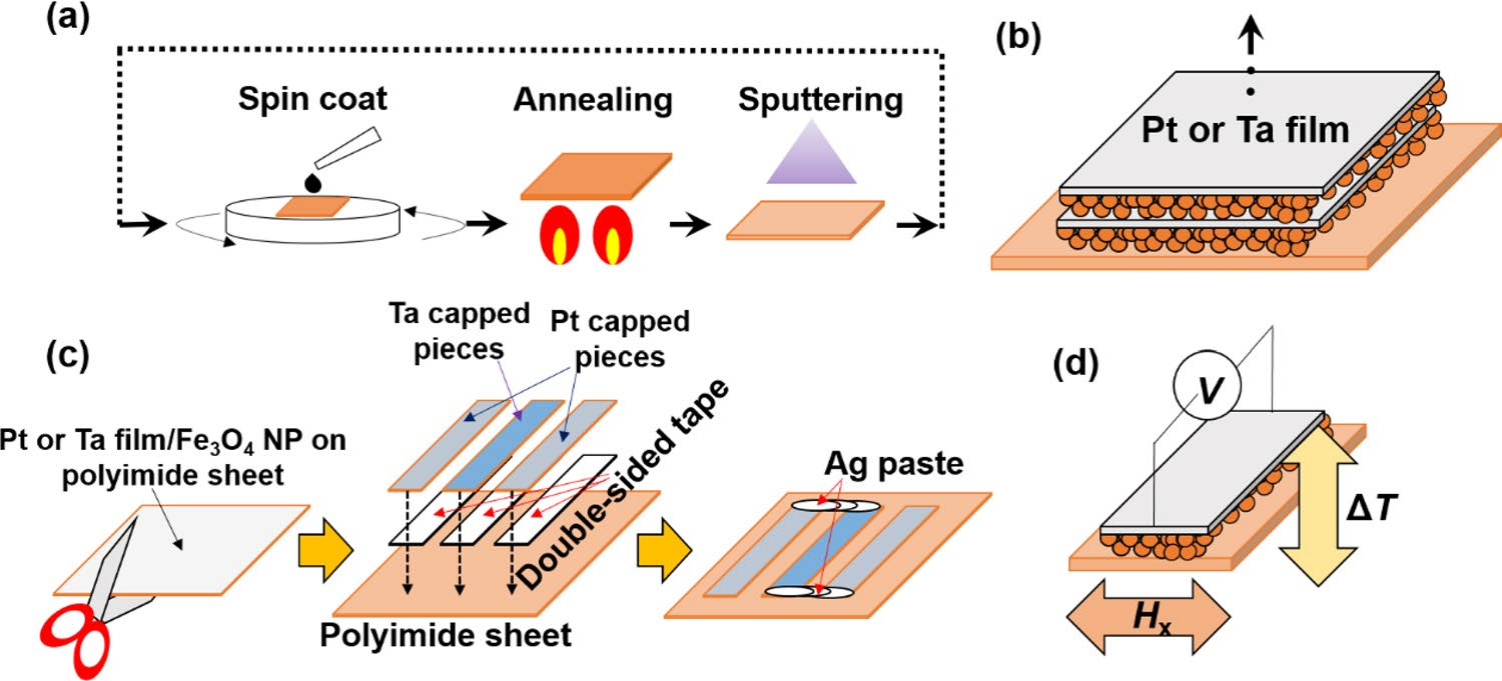
(**a**) Schematic of fabrication process of [Pt or Ta film/Fe_3_O_4_ nanoparticle]_*n*_ multilayered film, (**b**) [Pt or Ta film/Fe_3_O_4_ nanoparticle]_*n*_ multilayered film, (**c**) fabrication process of thermopile device using [Ta and Pt film/Fe_3_O_4_ nanoparticle] films on a polyimide sheets, and (**d**) spin Seebeck voltage measurement.

We used commercially available 20-nm-diameter Fe_3_O_4_ NPs, with the surface ligand of oleic acid in the toluene (IO-O20-50, Cytodiagnostics Inc.). The Fe_3_O_4_ NP-assembled films were fabricated on a 0.38-mm-thick thermally oxidized Si substrate or a 0.1-mm-thick polyimide sheet using spin coating. Then, to remove the solvent, the NP-assembled films were annealed in a vacuum at *T*_A_ = 373 K, 473 K, and 673 K. It was confirmed by SEM observation that the average Fe_3_O_4_ NP size does not change after annealing. Subsequently, a 5-nm-thick Pt or 8-nm-thick Ta or Ru film was deposited on top of the NP-assembled films by DC magnetron sputtering. The SSETEG output is significantly improved by laminating the magnetic and non-magnetic layers^10^. Based on this report, the [Pt or Ta film/Fe_3_O_4_ NP]_*n*_ multilayer films were fabricated by repeating the same process, where the number of stacking *n* was changed from 1 to 5, as shown in Fig. 1a,b. The dimension of samples for the SSE voltage measurement is 25 × 10 mm. Additionally, we fabricate thermopile devices using cut Ta or Pt capped Fe_3_O_4_ NP-assembled films on the polyimide sheet, shown in Fig. 1c. The dimension of cut NP-assembled films is 30 × 5 mm. Magnetic properties were measured by vibrating sample magnetometer (VSM). Sample morphologies were observed with a scanning electron microscope (SEM) and an atomic force microscopy (AFM). The chemical state of the NP-assembled films were measured by Fourier Transform Infrared Spectroscopy (FT-IR). The SSE voltage measurements were performed as follows. First, the temperature gradient was applied by sandwiching a sample using a pair of Peltier modules, and the temperature difference Δ*T* between the top and bottom surfaces of the substrate was monitored, as shown in Fig. 1d. Furthermore, the SSE voltage was measured while a magnetic field was swept between − 300 and 300 mT. The distance between voltage terminals *L*_y_ was fixed at 20 mm. For SSE voltage measurements, the transverse voltage $V_{{\text{T}}}$ can be expressed as $V_{{\text{T}}} = V_{{\text{O}}} + V_{{\text{S}}}$, where $V_{{\text{O}}}$ is an ordinary Nernst voltage generated from Pt, Ta, and those naturally oxidized layers, and $V_{{\text{s}}}$ is an SSE voltage. $V_{{\text{s}}}$ can be determined by subtracting the linear *H* dependence of $V_{{\text{T}}}$ in the range of the oversaturation field because $V_{{\text{O}}}$ linearly depends on *H*.

## Results and discussion

### Structural and magnetic properties of Fe_3_O_4_ nanoparticle films

**Figure 2:**
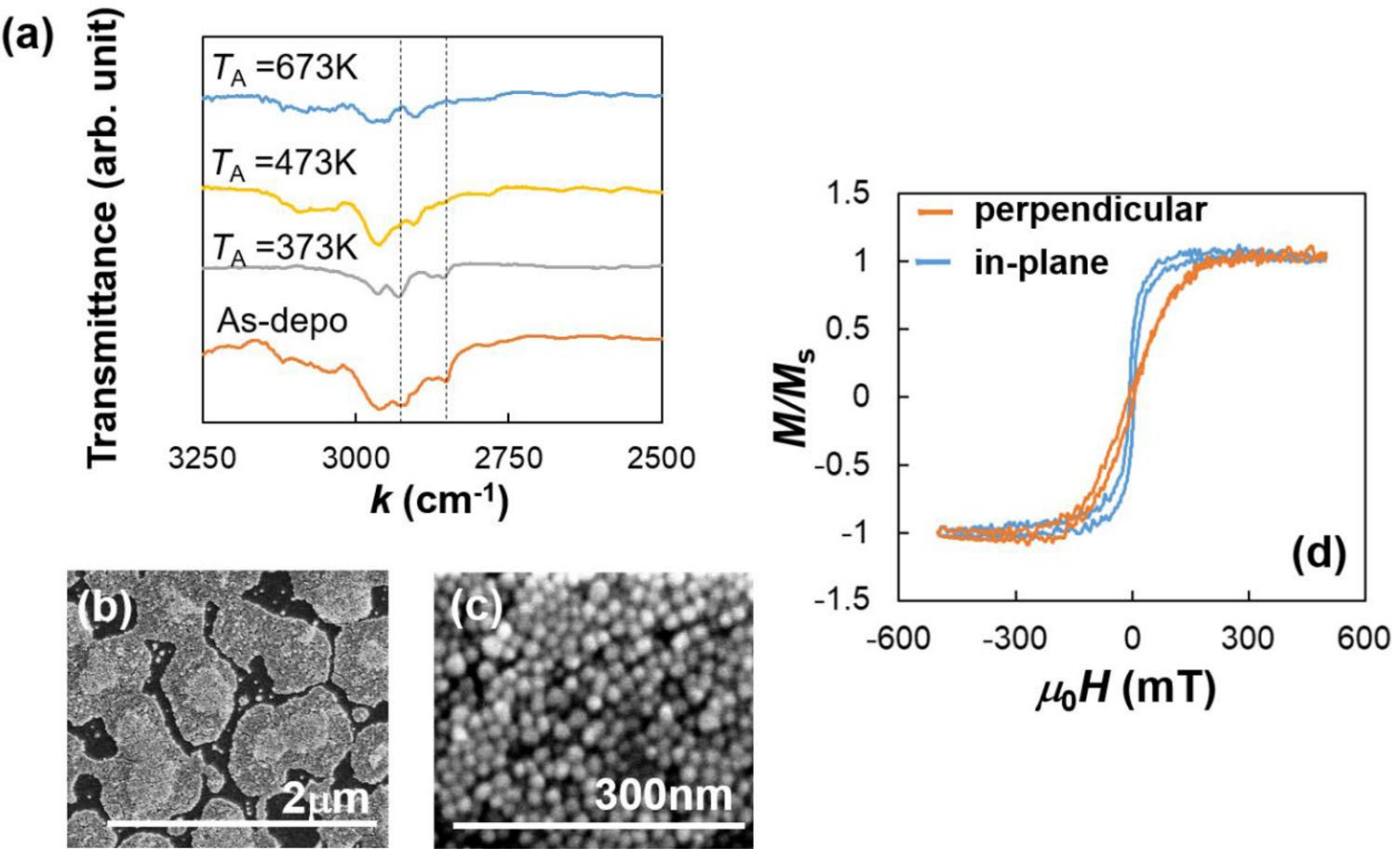
(**a**) FT-IR spectrums of Fe_3_O_4_ nanoparticle-assembled films on thermally oxidized Si substrate without or with annealing at *T*_A_ = 373, 473, and 673 K. Black broken lines indicate *k* = 2852 and 2922 cm^−1^. (**b**), (**c**) SEM images and (**d**) the normalized magnetization *M*/*M*_S_ curves of [Pt film/Fe_3_O_4_ nanoparticle]_1_ film on thermally oxidized Si substrate annealed at *T*_A_ = 473 K. The orange and blue lines in (**d**) were obtained under perpendicular and in-plane magnetic fields *H*, respectively.

Figure 2a indicates the IR transmittance as a function of wave number *k* for the Fe_3_O_4_ NP-assembled films on thermally-oxidized Si substrate as-deposited and annealed at *T*_A_ = 373, 473, and 673 K. The bands at *k* = 2852 and 2922 cm^−1^ were attributed to the asymmetric CH_2_ stretch and symmetric CH_2_ stretch in oleic acid^34^. The absorption at above bands appeared in the FR-IR spectrum for the Fe_3_O_4_ NP-assembled film annealed at *T*_A_ = 373 K and as-deposited, whereas they were absent in the spectrum of the film annealed at *T*_A_ = 473 K and 673 K. These results indicate that the surface ligands of NPs were decomposed when films were annealed at *T*_A_ $\ge$ 473 K. The decomposition of surface ligands is important for the SSETEG. Because the SSE voltage is generated in the interface between magnetic material and HM layer, the HM and magnetic layers should be contacted directly. Therefore, we fix *T*_A_ to 473 K hereafter, which is a sufficiently low temperature for a plastic sheet. Additionally, the sufficiently low *T*_A_ can avoid the interfacial atom diffusion and the contamination of SSE through an anomalous Nernst effect (ANE) of the intermixing layer. In fact, Pt-Fe alloying at the Fe_3_O_4_/Pt interface with high-temperature sputtering (~ 753 K) has been reported and ANE in the intermixing layer has been observed^35,36^. Figure 2b,c show SEM images of [Pt film/Fe_3_O_4_ NP]_1_ film on the thermally-oxidized Si substrate annealed at *T*_A_ = 473 K. As shown in Fig. 2b, NPs formed patchy patterns while remaining not coarsening and maintaining nanometer size with low thermal conductivity, as shown zoomed SEM image in Fig. 2c. Here, Fe_3_O_4_ NP-assembled films annealed at *T*_A_ = 473 K without Pt layer have insulating electric conductance, meaning NPs have large interface resistance attributed to a large number of interfaces between NPs. Figure 2d shows the in-plane and perpendicular magnetization curves of the [Pt film/Fe_3_O_4_ NP]_1_ film on thermally oxidized Si substrate annealed at *T*_A_ = 473 K, which indicates that the NP-assembled film has in-plane magnetic anisotropy. Considering the fact that the Fe_3_O_4_ NPs have spherical shape, the in-plane magnetic anisotropy is not caused by the shape anisotropy, indicating that the Fe_3_O_4_ NPs are magnetically connected and have film-like magnetic properties.

### Surface structure of multilayered Fe_3_O_4_ nanoparticle films

**Figure 3:**
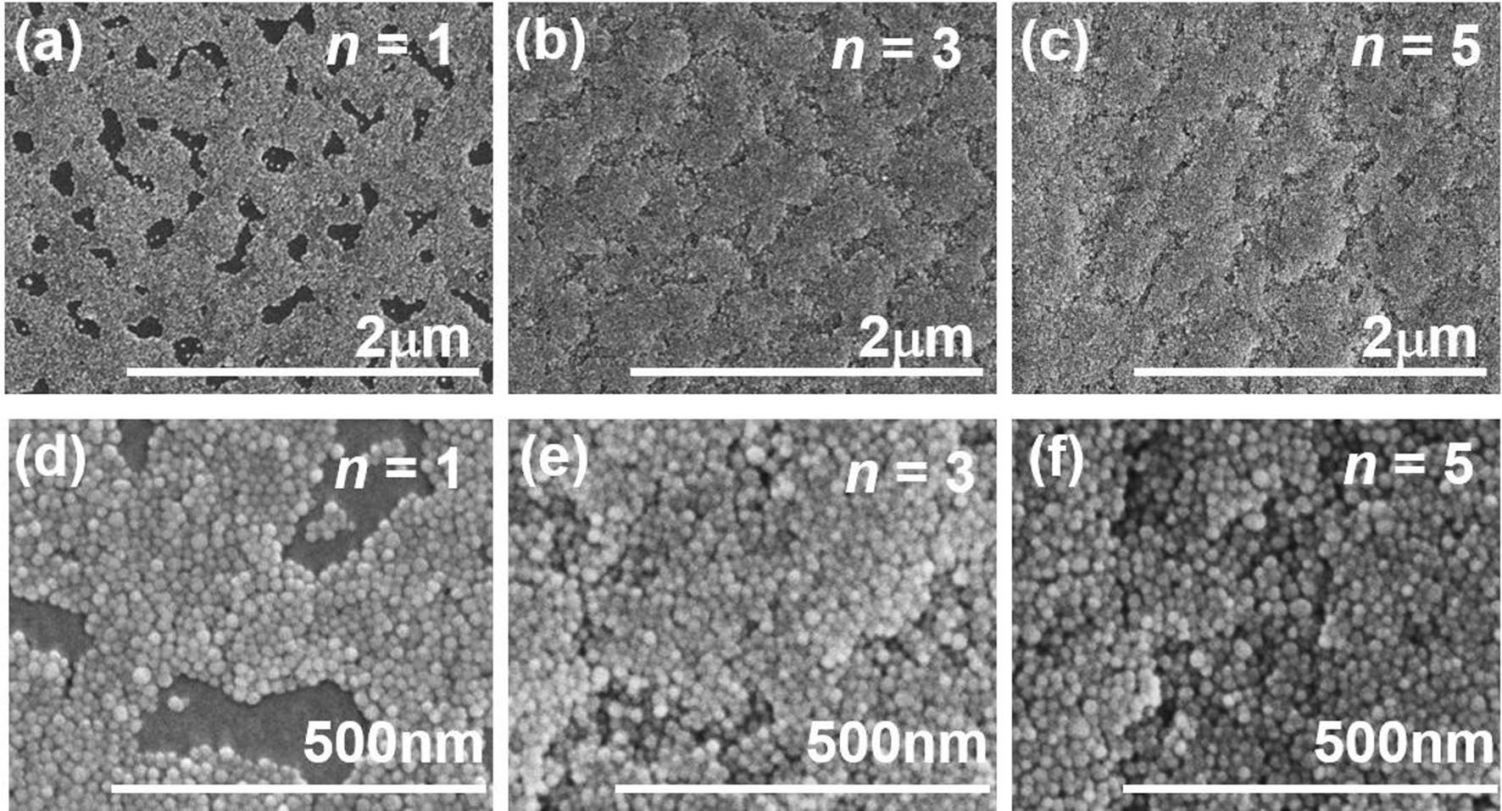
SEM images of the [Pt film/Fe_3_O_4_ nanoparticle]_*n*_ films on the polyimide sheet annealed at *T*_A_ = 473 K with (**a**) *n* = 1, (**b**) *n* = 3, and (**c**) *n* = 5. (**d**), (**e**), and (**f**) are enlarged SEM images of (**a**), (**b**), and (**c**), respectively.

**Figure 4:**
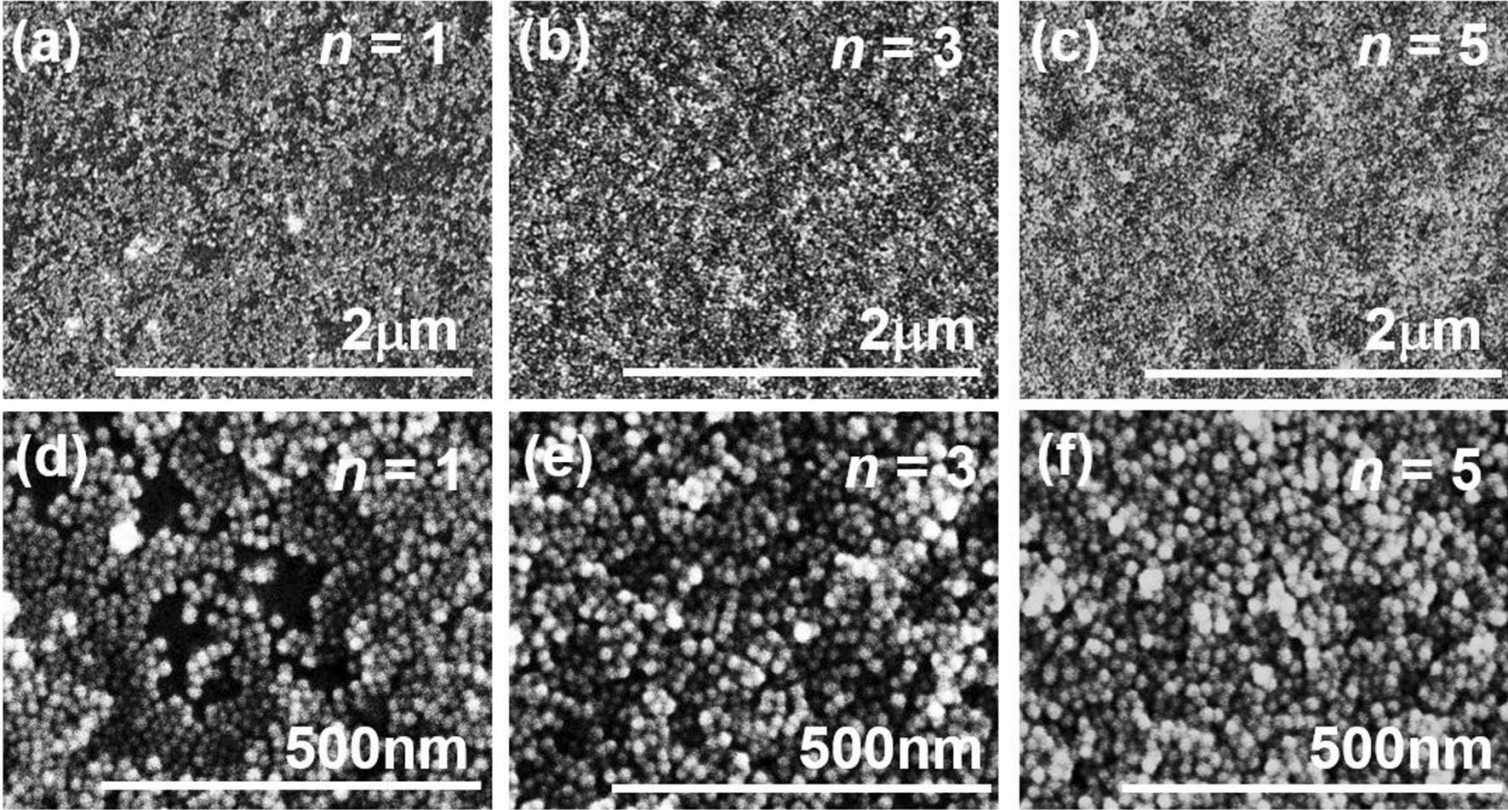
SEM images of the [Ta film/Fe_3_O_4_ nanoparticle]_*n*_ films on the polyimide sheet annealed at *T*_A_ = 473 K with (**a**) *n* = 1, (**b**) *n* = 3, and (**c**) *n* = 5. (**d**), (**e**), and (**f**) are enlarged SEM images of (**a**), (**b**), and (**c**), respectively.

Figure 3 shows SEM images of the [Pt film/Fe_3_O_4_ NP]_*n*_ films on the polyimide sheet with *n* = 1, 3, and 5. The SEM image of the [Pt film/Fe_3_O_4_ NP]_1_ film shows that the NP-assembled film does not completely cover the polyimide sheet. On the other hand, the [Pt film/Fe_3_O_4_ NP]_*n*_ film with *n* = 3 and 5 cover the polyimide sheet, completely. Figure 4 shows SEM images of the [Ta film/Fe_3_O_4_ NP]_*n*_ films on the polyimide sheet with *n* = 1, 3, and 5. The SEM image of the [Ta film/Fe_3_O_4_ NP]_1_ film also shows that the NP-assembled film does not completely cover the polyimide sheet and the [Ta film/Fe_3_O_4_ NP]_*n*_ film with *n* = 3 and 5 cover the polyimide sheet, completely.

**Figure 5:**
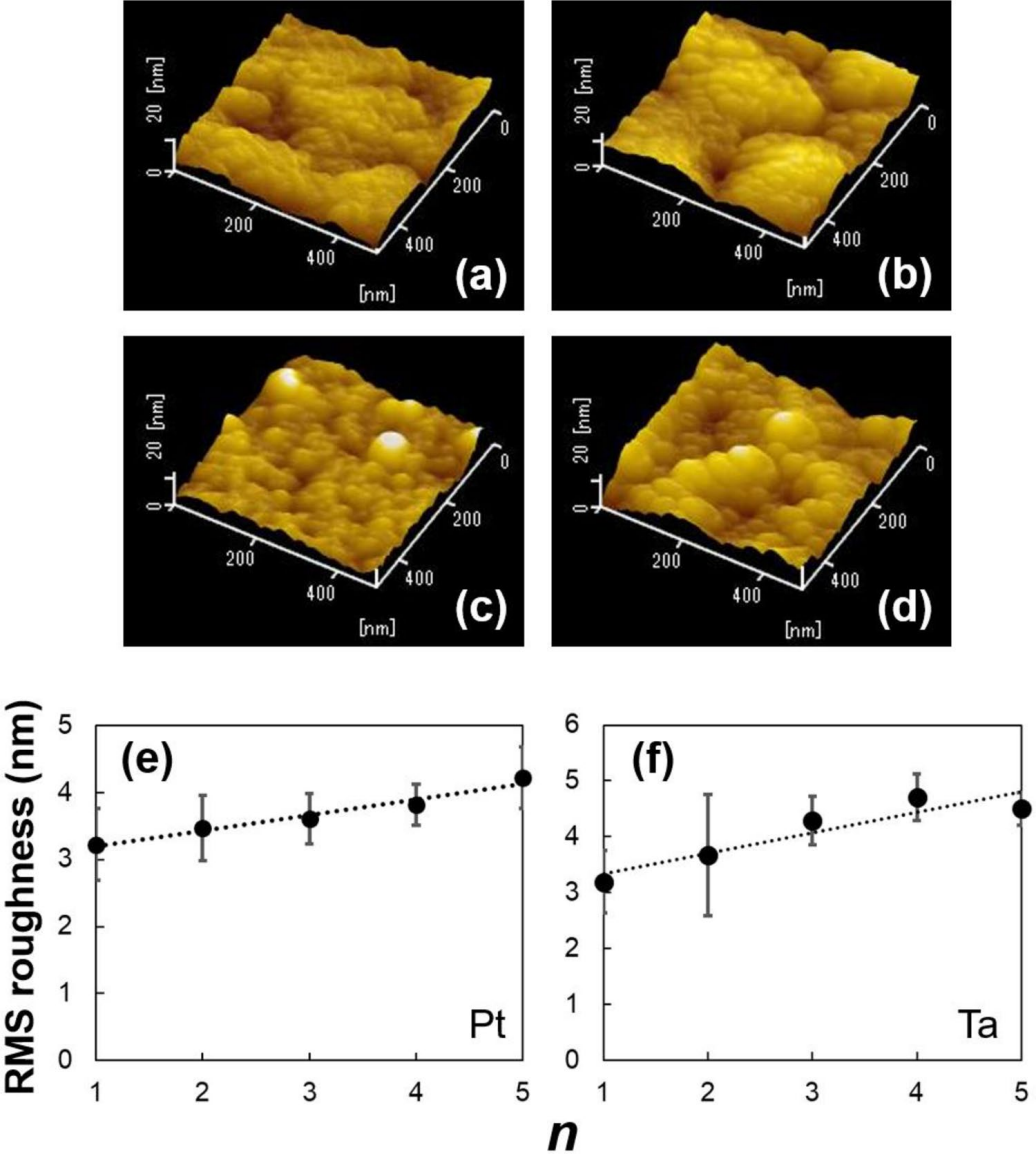
AFM images of the [Pt film/Fe_3_O_4_ nanoparticle]_*n*_ films with (**a**) *n* = 1 and (**b**) *n* = 5, and the [Ta film/Fe_3_O_4_ nanoparticle]_*n*_ films with (**c**) *n* = 1 and (**d**) *n* = 5 on the polyimide sheet annealed at *T*_A_ = 473 K. Root mean square roughness as a function of number of stacking *n* for Fe_3_O_4_ nanoparticle-assembled films with (**e**) Pt and (**f**) Ta layers. The black dash lines were estimated by least-squares method.

Figures 5a,d show the AFM images of [Pt or Ta film/Fe_3_O_4_ NP]_*n*_ films with *n* = 1 and 5 on the polyimide sheet. The small periodic roughness caused by NPs were visible in all samples. In addition to this, the long periodic roughness because of the accumulation of NPs can be seen in only [Pt film/Fe_3_O_4_ NP]_*n*_ films. It is well known that the morphology of NPs is strongly affected by surface energy^37,38^. In the case of Fe_3_O_4_ NP multilayered films, the surface energy of oxidized Ta or Pt probably affects the morphology of NP-assembled films. The surface of Ta was highly oxidized under ambient air, while that of Pt was not oxidized, which is explained by the fact that the surface energy *γ* of Pt (*γ* ~ 2.5 J/m^2^) is greatly larger than that of Ta_2_O_5_ (*γ* ~ 0.07 J/m^2^)^39,40^. Therefore, the difference of surface energy is one of the factors for the difference of morphology. However, further investigation is required to reveal the morphology of Fe_3_O_4_ NP multilayered films. Figure 5e,f show the root mean square (RMS) roughness of [Pt or Ta film/Fe_3_O_4_ NP]_*n*_ films on the polyimide sheet. Both of the RMS roughness of Fe_3_O_4_ NP multilayered films with the Pt and Ta layers increase with increasing *n*.

### Spin Seebeck measurements for Fe_3_O_4_ nanoparticle-assembled films

**Figure 6:**
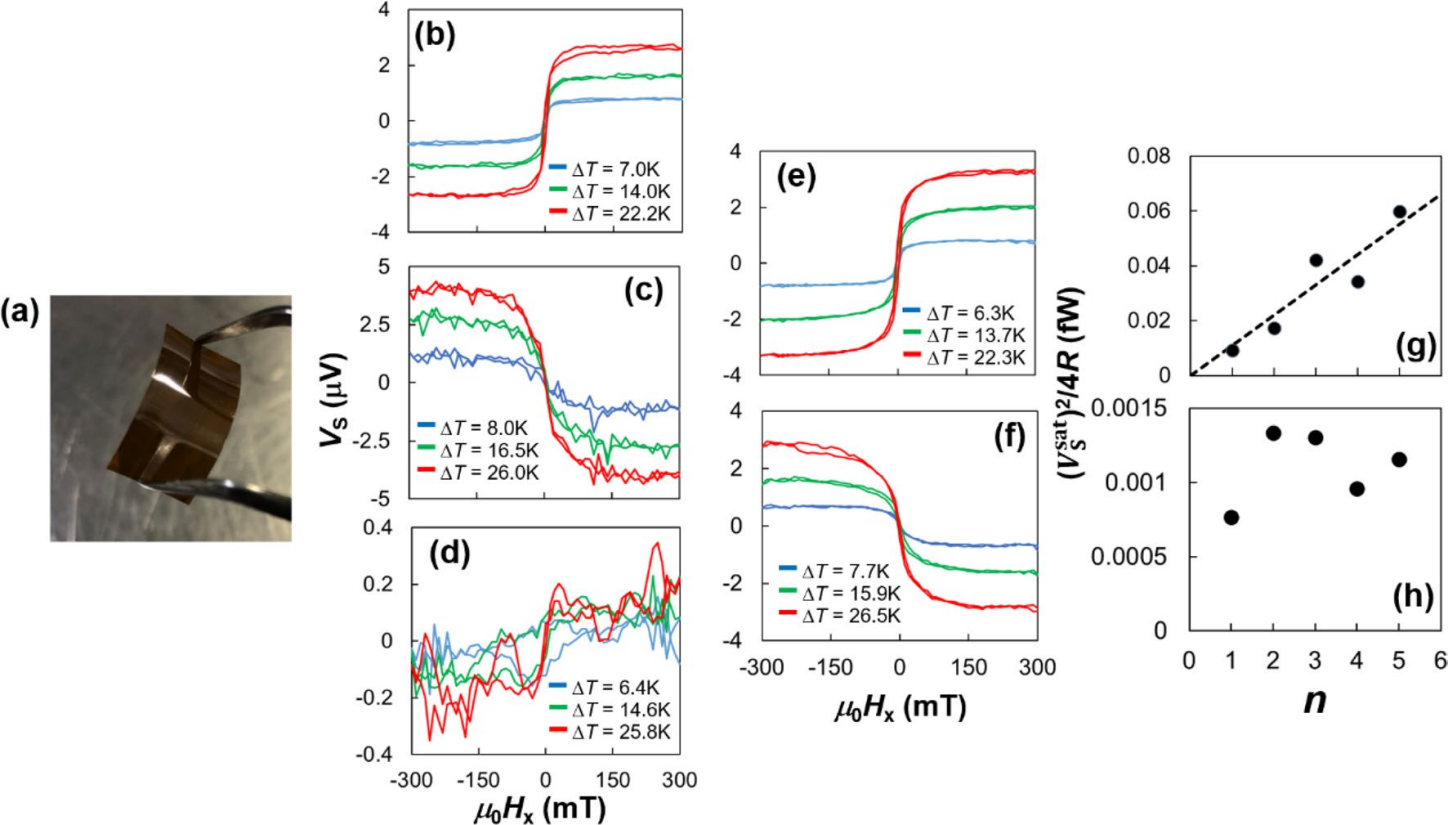
(**a**) Photograph of the [Pt film/Fe_3_O_4_ nanoparticle]_1_ film on the polyimide sheet annealed at *T*_A_ = 473 K. The spin Seebeck voltage *V*_S_ as a function of in-plane magnetic field *H*_x_ under various temperature difference Δ*T* for (**b**) [Pt film/Fe_3_O_4_ nanoparticle]_1_, (**c**) [Ta film/Fe_3_O_4_ nanoparticle]_1_, (**d**) [Ru film/Fe_3_O_4_ nanoparticle]_1_ (**e**) [Pt film/Fe_3_O_4_ nanoparticle]_5_, and (**f**) [Ta film/Fe_3_O_4_ nanoparticle]_5_ films, where films were annealed at *T*_A_ = 473 K. The ideal maximum thermoelectric power $V_{{\text{S}}}^{{{\text{sat2}}}}$/4*R* as a function of the number of stacking *n* for (**g**) [Pt film/Fe_3_O_4_ nanoparticle]_*n*_ and (**h**) [Ta film/Fe_3_O_4_ nanoparticle]_*n*_ films with annealing at *T*_A_ = 473 K. Black broken line in the (**g**) is estimated by least‐squares method.

Figure 6a shows the photograph of the [Pt film/Fe_3_O_4_ NP]_1_ film on polyimide sheet annealed at *T*_A_ = 473 K. The assembled film has sufficient flexibility. Figure 6b,c show the *V*_S_ of [Pt or Ta film/Fe_3_O_4_ NP]_1_ film as a function of the *H*_x_ under various Δ*T*. *V*_S_ loops clearly appear and it strongly depend on Δ*T*. Additionally, the polarity of *V*_S_ loops in Fig. 6b,c are opposite to each other. In the case of the SSETEG, the polarity of *V*_S_ loop strongly depends on spin Hall angle in heavy metal (HM) layer because the magnon spin current is converted into the electric current by the ISHE in the HM layer, whose strength and polarity are determined by spin Hall angle^5,20,22,32^. Moreover, the generated *V*_S_ changed in response to magnetization change shown in Fig. 2d. Since the direction of spin current is determined by the direction of magnetization in magnetic layer, the thermoelectric voltage is generated by the SSE in the Fe_3_O_4_ NP-assembled films as the conventional SE does not depend on magnetization. If the NPs are completely covered by oleic acid, the magnon spin current in the NPs cannot flow into the HM layer. The finite thermoelectric voltage showed that the HM layer and NPs are directly in contact with each other, which is supported by the FT-IR analysis shown in Fig. 2a. However, there is a possibility that the finite residual oleic acid and/or hydrocarbons generated from decomposition of oleic acid were still on the NPs, whereas the SSE voltages were clearly observed. Recently, spin currents flowing in the organic materials have been reported^41,42^. Therefore, we expected that the spin current can also flow in oleic acid and/or hydrocarbons. However, the almost all spin current flow into HM layer directly through the bared surface of NPs.

Although the polarities of SSE in the Fe_3_O_4_ NP-assembled films reflect the spin Hall angle of the HM layer, the observed *V*_S_ possibly has a finite ANE component as ANE of the metallic Fe_3_O_4_ film has been previously reported^7^. To observe the ANE component through Fe_3_O_4_ NPs, the [Ru film/Fe_3_O_4_ NP]_1_ film, where Ru has a negligibly small spin Hall angle^43^, was fabricated. Figure 6d shows the *V*_S_ of [Ru film/Fe_3_O_4_ NP]_1_ film as a function of *H*_x_ under various Δ*T*. The *V*_S_ of the [Ru film/Fe_3_O_4_ NP]_1_ film is much smaller than that of the [Pt or Ta film/Fe_3_O_4_ NP]_1_ film. The $V_{{\text{S}}}^{{{\text{sat}}}} / \Delta T$ value of the [Ru film/Fe_3_O_4_ NP]_1_ film, where $V_{{\text{S}}}^{{{\text{sat}}}}$ is the saturated *V*_S_ in the sufficiently large *H*_x_, is 0.007 µV/K, which is almost 17 times smaller than that of the [Pt film/Fe_3_O_4_ NP]_1_ film, as shown below. Therefore, we conclude that the ANE component of Fe_3_O_4_ NPs is negligibly smaller than that of SSE. Additionally, the Fe_3_O_4_ NP-assembled film has large resistivity *ρ* > 0.2 Ωm, which is much larger than that of the metallic Fe_3_O_4_ film (*ρ* = 5 × 10^−5^ Ωm)^7^. The ANE component on the Fe_3_O_4_ NP-assembled film would be strongly suppressed by the metallic Pt or Ta layer because of the large *ρ* value of the Fe_3_O_4_ NP-assembled film. Therefore, the *V*_S_ in the [Pt or Ta film/Fe_3_O_4_ NP]_1_ film is dominated by the SSE.

To compare the magnitude of SSE voltage, $V_{{\text{S}}}^{{{\text{sat}}}} / \Delta T$ values were estimated. The $V_{{\text{S}}}^{{{\text{sat}}}} / \Delta T$ of [Pt film/Fe_3_O_4_ NP]_1_ film is 0.12 µV/K, which is smaller than that of epitaxial Fe_3_O_4_ film ($V_{{\text{S}}}^{{{\text{sat}}}} / \Delta T = 1.2$µV/K)^7^. One of the reasons for the reduction in $V_{{\text{S}}}^{{{\text{sat}}}} / \Delta T$ is the low thermal conductivity of the flexible polyimide sheet. Strictly speaking, the $V_{{\text{S}}}^{{{\text{sat}}}}$ depends on the temperature difference in Fe_3_O_4_ NP layer $\Delta T_{{{\text{Fe}}_{3} {\text{O}}_{4} }}$, which determined by difference of the temperature between the top and bottom surface of Fe_3_O_4_ NP layer. The substrate with low thermal conductivity decreases the $\Delta T_{{{\text{Fe}}_{3} {\text{O}}_{4} }}$. The thermal conductivity of polyimide and SrTiO_3_, which was used as substrate in epitaxial Fe_3_O_4_ film, are 0.2 W/mK and 10 W/mK, respectively^44,45^. Therefore, we estimated the spin Seebeck coefficient *S*_SSE_, which does not depend on the sample size and substrate material. However, the estimation of $\Delta T_{{{\text{Fe}}_{3} {\text{O}}_{4} }}$, which is used to determine *S*_SSE_, is complicated because the Fe_3_O_4_ NP layer is much thinner than the substrate material. There are several methods to estimate a sample independent of the spin Seebeck coefficient without determining the $\Delta T$ of the thin magnetic layer; for example, the heat flux normalized spin Seebeck coefficient^46^. In this study, to estimate *S*_SSE_, we calculated a temperature gradient for Fe_3_O_4_ NP-assembled film $\nabla T_{{{\text{Fe}}_{3} {\text{O}}_{4} }}$ using the following equation^47^:

1 $$ \nabla T_{{{\text{Fe}}_{3} {\text{O}}_{4} }} = \frac{{\kappa_{{{\text{polyimide}}}} }}{{\kappa_{{{\text{Fe}}_{3} {\text{O}}_{4} }}^{{\text{N}}} }}\nabla T_{{{\text{polyimide}}}} $$

where $\kappa_{{{\text{polyimide}}}}$ and $\kappa_{{{\text{Fe}}_{3} {\text{O}}_{4} }}^{{\text{N}}}$ are the thermal conductivity of polyimide and Fe_3_O_4_ NP-assembled films, respectively. When the thickness of the Fe_3_O_4_ NP-assembled film is much smaller than that of the polyimide sheet, the temperature gradient in the polyimide sheet $\nabla T_{{{\text{polyimide}}}}$ can be represented as $\nabla T_{{{\text{polyimide}}}} = \Delta T/L_{{\text{z}}}^{{{\text{polyimide}}}}$, where $L_{{\text{z}}}^{{{\text{polyimide}}}} = { }0.1{\text{ mm}}$ is the thickness of the polyimide sheet. The thermal conductivity of the bulk Fe_3_O_4_ $\kappa_{{{\text{Fe}}_{3} {\text{O}}_{4} }}^{{\text{B}}}$ has been estimated as 3.8 W/mK^48^. The $\kappa_{{{\text{Fe}}_{3} {\text{O}}_{4} }}^{{\text{N}}}$ should be reduced from $\kappa_{{{\text{Fe}}_{3} {\text{O}}_{4} }}^{{\text{B}}}$ because the NP-assembled film has numerous voids. To roughly estimate $\kappa_{{{\text{Fe}}_{3} {\text{O}}_{4} }}^{{\text{N}}}$, we assumed that $\kappa_{{{\text{Fe}}_{3} {\text{O}}_{4} }}^{{\text{N}}}$ can be represented as $\kappa_{{{\text{Fe}}_{3} {\text{O}}_{4} }}^{{\text{N}}} = f\kappa_{{{\text{Fe}}_{3} {\text{O}}_{4} }}^{{\text{B}}}$, where *f* is the packing fraction of the NP-assembled film. Several studies, which investigated the *f* value in randomly deposited NP-assembled films^49–52^, have reported the estimated *f* value as ranging from 0.25 to 0.35. *S*_SSE_ is estimated with the following equation using $\nabla T_{{{\text{Fe}}_{3} {\text{O}}_{4} }}$ instead of ${\Delta }T_{{{\text{Fe}}_{3} {\text{O}}_{4} }}$:

2 $$ S_{{{\text{SSE}}}} = \frac{{V_{{\text{S}}} }}{{\nabla T_{{{\text{Fe}}_{3} {\text{O}}_{4} }} L_{{\text{y}}} }}{ } $$

where *L*_y_ is 20 mm. When *f* is 0.30, *S*_SSE_ of the [Pt film/Fe_3_O_4_ NP]_1_ film is 3.4 nV/K, as shown in Table 1. *S*_SSE_ of the epitaxial Fe_3_O_4_ film with the Pt layer has been estimated to be 74 nV/K^7^, which is 22 times larger than that of the [Pt film/Fe_3_O_4_ NP]_1_ film. There are two possible reasons for reduced *S*_SSE_. The first reason is the boundary scattering of the phonons and magnons. The SSE voltage is reduced when the grain size is much smaller than the mean free paths of magnons and phonons^53^. In the present work, the Fe_3_O_4_ NPs have 20 nm diameter, which is much smaller than that of the epitaxial Fe_3_O_4_ film. Therefore, the small Fe_3_O_4_ NPs can reduce the *S*_SSE_. The second reason is the presence of residual oleic acid and/or hydrocarbons on the surface of NPs. As mentioned above, unclean surfaces of NPs result in strong scattering of the magnon spin current induced by $\Delta T$, which can lead to a reduction in *S*_SSE_.

**Table 1:** Comparisons of resistance *R*, $V_{{\text{S}}}^{{{\text{sat}}}} /\Delta T$, and *S*_SSE_ of [Pt or Ta film/Fe_3_O_4_ NP]_*n*_ films used in spin Seebeck voltage measurement.

HM film	Parameter	*n* = 1	*n* = 2	*n* = 3	*n* = 4	*n* = 5
Pt	*R* (Ω)	381	224	135	118	94
	$V_{{\text{s}}}^{{{\text{sat}}}} / \Delta T$(µV/K)	0.12	0.12	0.15	0.13	0.15
	*S*_SSE_ (nV/K)	3.4	3.4	4.3	3.7	4.3
Ta	*R* (Ω)	8020	3740	2450	3150	2330
	$V_{{\text{s}}}^{{{\text{sat}}}} / \Delta T$(µ/K)	− 0.16	− 0.14	− 0.12	− 0.11	− 0.10
	*S*_SSE_ (nV/K)	− 4.6	− 4.0	− 3.4	− 3.1	− 2.9

Figure 6e,f show the *V*_S_ of [Pt or Ta film/Fe_3_O_4_ NP]_5_ films as a function of the *H*_x_ under various Δ*T*. These exhibit clear *V*_S_ loops with the same shape as those of samples for *n* = 1. Here, the $V_{{\text{S}}}^{{{\text{sat}}}} / \Delta T$ of [Pt film/Fe_3_O_4_ NP]_*n*_ with *n* = 1 and 5 are 0.12 µV/K and 0.15 µV/K, respectively. As shown in Table 1, we found that the $V_{{\text{S}}}^{{{\text{sat}}}} / \Delta T$ was almost unchanged by increasing *n*, whereas the resistance *R* decreases with increasing *n*. In contrast, in the case of Fe_3_O_4_ NP-assembled films with Ta cap layer, the $\left| {V_{{\text{S}}}^{{{\text{sat}}}} / \Delta T} \right|$ was slightly decreased by increasing *n*. Additionally, *R* of Ta capped samples are not monotonically decreased by increasing *n*, while *R* decreases from 381 to 94 Ω in Pt capped samples with increasing *n*. These details are shown in the Table 1. This is associated with the fact that Ta is easily oxidized in the ambient air, whereas Pt does not oxidize. To clarify the resistivity effect on the SSE performance, generated powers are derived in the following.

Here, the output thermoelectric power *P*_O_, using a load resistance *R*_L_ connected to SSE device as serial circuit, should be estimated as *P*_O_ = *V*_L_^2^/*R*_L_, where *V*_L_ is the voltage on *R*_L_. Additionally, when a contact resistance is small enough, the ideal maximum output thermoelectric power *P*_ideal_ can be expressed as $V_{{\text{S}}}^{{{\text{sat2}}}}$/4*R* instead of the maximum *P*_O_^54^*.* In this study, for the sake of simplicity, we use *P*_ideal_ as a thermoelectric power of SSETEG. Figure 6g shows the $V_{{\text{S}}}^{{{\text{sat2}}}}$/4*R* at Δ*T* = 1 K of [Pt film/Fe_3_O_4_ NP]_*n*_ films as a function of *n*. The thermoelectric power linearly increases with *n*. It indicates that when *n* increases, $V_{{\text{S}}}^{{{\text{sat}}}} / \Delta T$ remains almost constant, whereas *R* decreases as mentioned above. Here, because the *V*_S_ depends on temperature gradient $\nabla T_{{{\text{Fe}}_{3} {\text{O}}_{4} }}$ in Fe_3_O_4_ NPs film, there is a possibility that $\nabla T_{{{\text{Fe}}_{3} {\text{O}}_{4} }}$ decreases with an increase in the total thickness $t_{{{\text{Fe}}_{3} {\text{O}}_{4} }}$ under the fixed Δ*T* between the top and bottom surfaces, leading to a smaller power than expected from the low *R*. Therefore, Fig. 6g indicates that the $\nabla T_{{{\text{Fe}}_{3} {\text{O}}_{4} }}$ can be regarded as an independent value with respect to $t_{{{\text{Fe}}_{3} {\text{O}}_{4} }}$ because the $t_{{{\text{Fe}}_{3} {\text{O}}_{4} }}$ is significantly smaller than the thickness of the polyimide sheet. In the case of ANE, the thickness independence of ANE voltage *V*_ANE_ of the FePt film have been reported^6^. It is note that the ANE and SSE have similar physical aspects because the electric or magnon spin current is generated by Δ*T*, and it is converted into electric current by the scattering by the magnetization (ANE) or ISHE (SSE). Therefore, our results are plausible. Additionally, the enhancement of spin Seebeck voltage in [Pt/Fe_3_O_4_]_*n*_ multilayer with $n \le 6$ has been reported^10^. According to the report, when temperature difference was applied in the thickness direction, the spin current flowing between Fe_3_O_4_ layer in that direction enhanced. In our case, the average spin current in the multilayered Fe_3_O_4_ NP-assembled film can also be enhanced, which leads to *n-*independence of $V_{{\text{S}}}^{{{\text{sat}}}} / \Delta T$. However, the $V_{{\text{S}}}^{{{\text{sat}}}} / \Delta T$ of [Pt film/Fe_3_O_4_ NP]_*n*_ films did not increase with increasing *n*, whereas that of the [Pt/Fe_3_O_4_]_*n*_ multilayered film increased^10^. It was also reported that the $V_{{\text{S}}}^{{{\text{sat}}}} / \Delta T$ remains unchanged, while the *R* value decreases in the case of a [Pt/YIG]_*n*_ multilayered film; this was attributed to the difference in the interface structure between the highly epitaxial [Pt/Fe_3_O_4_]_*n*_ and the non-epitaxial [Pt/YIG]_*n*_^55^. In the present study, interfaces between Fe_3_O_4_ NPs and Pt film are not epitaxial; additionally, there is a possibility that the finite residual oleic acid and/or hydrocarbons were still present on the surface of NPs. Thus, the $V_{{\text{S}}}^{{{\text{sat}}}} / \Delta T$ did not increase, although the lamination number *n* is increased in [Pt film/Fe_3_O_4_ NP]_*n*_ films. However, these results indicate that the thermoelectric power can be enhanced by increasing *n* as long as the total thickness of [Pt film/Fe_3_O_4_ NP]_*n*_ films remains much smaller than that of polyimide sheet. In contrast, the $V_{{\text{S}}}^{{{\text{sat2}}}}$/4*R* at Δ*T* = 1 K of the [Ta film/Fe_3_O_4_ NP]_*n*_ films was not increased with increasing *n*, as shown in Fig. 6h. One of the reasons for the trend of $V_{{\text{S}}}^{{{\text{sat2}}}} /4R$ of the [Ta film/Fe_3_O_4_ NP]_*n*_ films is the surface roughness. As mentioned above, the RMS roughness of [Pt or Ta film/Fe_3_O_4_ NP]_*n*_ films increase with increasing *n*. The surface roughness strongly affects the coverage of the film^56^. Large roughness promotes the generation of thinner sputtered layer because of geometrical shadowing. The ISHE in the much thin Ta layer existing partially is deactivated since it can be fully oxidized, and its electrical conductivity is lost. As a result, $V_{{\text{S}}}^{{{\text{sat}}}} / \Delta T$ can be reduced with increasing *n*, as shown in Table 1. Additionally, the roughness also affects the *R* values because the amount of fully oxidized Ta layer increases with increasing RMS roughness. However, the *R* of the [Ta film/Fe_3_O_4_ NP]_*n*_ films did not monotonically decrease with increasing *n*. It is because the amount of metal Ta layer also increased with increasing *n*. These lead to the irregular trend of thermoelectric power of the [Ta film/Fe_3_O_4_ NP]_*n*_ films against *n*.

### Spin Seebeck measurements for thermopile devices

**Figure 7:**
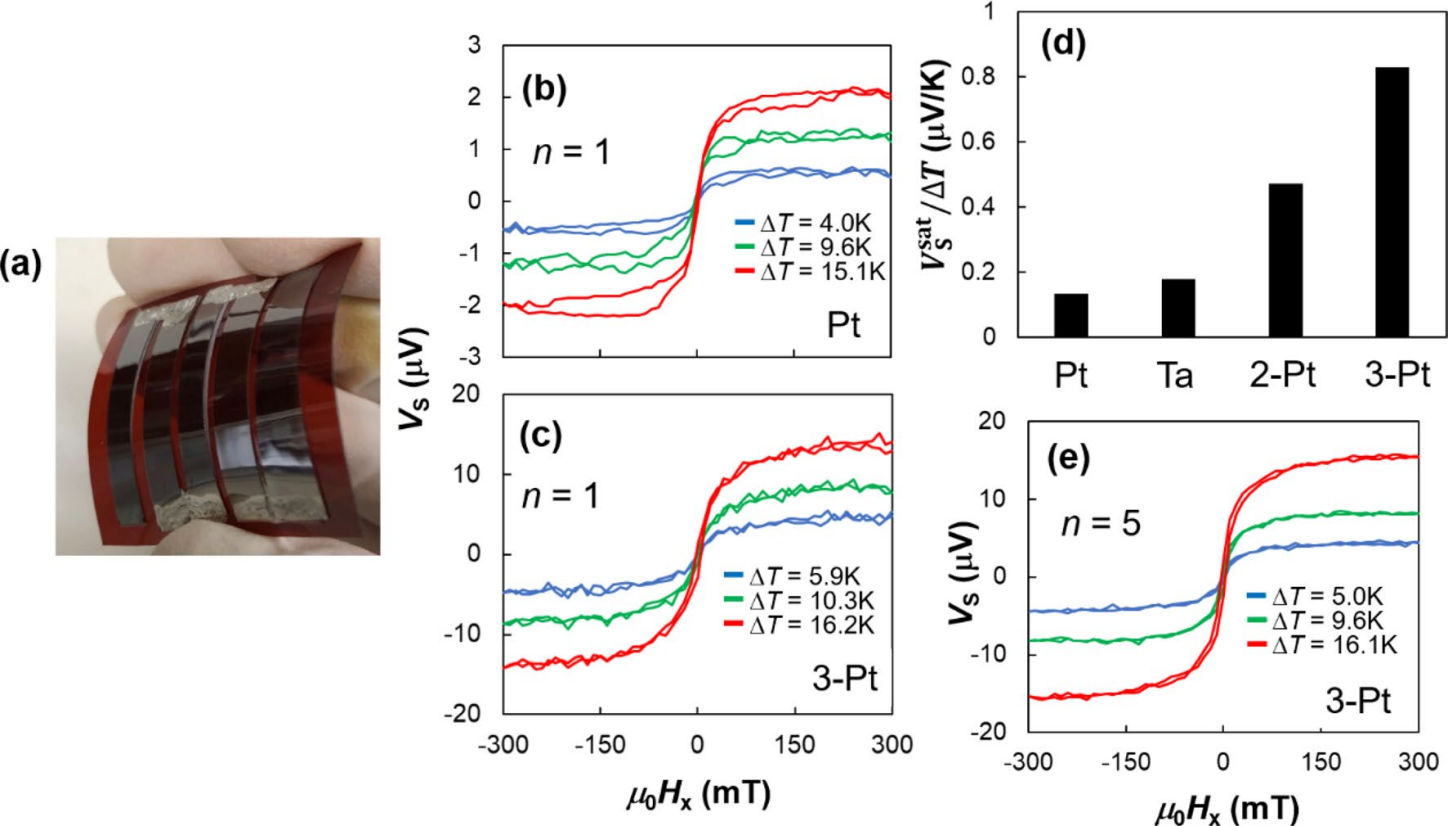
(**a**) Photograph of the 3-Pt thermopile device using [Pt or Ta film/Fe_3_O_4_ nanoparticle]_1_ pieces on the polyimide sheet annealed at *T*_A_ = 473 K. Spin Seebeck voltage *V*_S_ as a function of in-plane magnetic field *H*_x_ for (**b**) single [Pt film/Fe_3_O_4_ nanoparticle]_1_ piece and (**c**) 3-Pt thermopile device with [Pt or Ta film/Fe_3_O_4_ nanoparticle]_1_ pieces. (**d**) $V_{{\text{S}}}^{{{\text{sat}}}} / \Delta T$ for single [Pt and Ta film/Fe_3_O_4_ nanoparticle]_1_ pieces, 2-Pt and 3-Pt thermopile devices with *n* = 1. (**e**) Spin Seebeck voltage *V*_S_ as a function of in-plane magnetic field *H*_x_ for 3-Pt thermopile device with [Pt and Ta film/Fe_3_O_4_ nanoparticle]_5_ pieces.

Although the thermoelectric power is an essential performance, tuning of the thermoelectric voltage is also important in practical scenarios because the voltage originated from thermoelectric generation is typically low to operate the devices, for example light emitting diode needs a few volts. Even when the power is sufficient, a booster circuit will be needed. Therefore, to enhance the *V*_S_ at the same power, we fabricate a thermopile device using the series circuit consisting of SSETEG. Figure 7a shows the photograph of the thermopile device combined [Pt film/Fe_3_O_4_ NP]_1_ and [Ta film/Fe_3_O_4_ NP]_1_ pieces annealed at *T*_A_ = 473 K. The thermopile device maintains sufficient flexibility, although two polyimide sheets are stacked. Adjacent pieces are electrically connected using Ag paste. The thermopile device shown in Fig. 7a consists of two pairs of pieces, that is, [Pt film/Fe_3_O_4_ NP]_*n*_ and [Ta film/Fe_3_O_4_ NP]_*n*_, and one piece of [Pt film/Fe_3_O_4_ NP]_*n*_. Here, let us name the sample with *N* Pt-capped pieces “*N*-Pt thermopile device”, for instance 3-Pt thermopile device for the sample in Fig. 7a. Figure 7b,c show the *V*_S_ dependence on magnetic field for the single piece of [Pt film/Fe_3_O_4_ NP]_1_ and 3-Pt thermopile device with *n* = 1. The $V_{{\text{S}}}^{{{\text{sat}}}}$ of 3-Pt thermopile device is larger than that of the single piece because the 3-Pt thermopile device has five power sources originated from the five pieces. Figure 7d shows the $V_{{\text{S}}}^{{{\text{sat}}}} / \Delta T$ for the single pieces of 2-Pt and 3-Pt thermopile devices with *n* = 1. The $V_{{\text{S}}}^{{{\text{sat}}}} / \Delta T$ is clearly enhanced with. increasing the number *N* of Pt-capped pieces in series circuit. Therefore, we found that the *V*_S_ can be enhanced by connecting the cut [Ta and Pt film/Fe_3_O_4_ NP]_1_ piece. We note that the thermoelectric power of thermopile devices is not enhanced, whereas *V*_S_ is enhanced. It is because the resistance of thermopile devices increased due to series circuit of pieces. To enhance the thermoelectric power, the area of the SSE devices must be enlarged. However, the thermopile devices are useful for tuning the internal resistance according to the load resistance. This result represents a simple fabrication method for the thermopile device because we do not need to use lithography techniques, which is advantageous for widespread use.

Finally, we combined the multilayer Fe_3_O_4_ NP-assembled film and thermopile device. Figure 7e shows the *V*_S_ of the 3-Pt thermopile device with [Ta and Pt film/Fe_3_O_4_ NP]_5_ pieces. It has almost the same $V_{{\text{S}}}^{{{\text{sat}}}} / \Delta T$ as that with *n* = 1, whereas *R* should be decreased by thickening. Consequently, the estimated ideal maximum thermoelectric power $V_{{\text{S}}}^{{{\text{sat}}^{2} }} / 4R$ achieved 0.0160 fW, which is 5.5 times larger than that with *n* = 1 (0.0029 fW). Therefore, it was demonstrated that the thermopile device using multilayer NP-assembled film can realize both the high thermoelectric voltage and the high power.

According to the results of SSETEG using [Pt film/Fe_3_O_4_ NP]_5_ film and 3-Pt thermopile devices, the Fe_3_O_4_ NP-assembled film achieves high scalability and sufficient flexibility. We emphasize that Fe_3_O_4_ NPs can be easily deposited using spin coating, and the produced thin films can be formed into complex patterns without lithography. This simple fabrication method has potential applications for further acceleration of scalability.

## Conclusions

In summary, we fabricated the Fe_3_O_4_ NP-assembled films using spin coating capped Pt or Ta layer by a DC sputtering for spin Seebeck thermoelectric generator. FT-IR results indicate the surface ligand of NPs were broken when the NP-assembled films were annealed at *T*_A_ $\ge$ 473 K, which is an acceptable temperature for a plastic sheet. SEM images indicate that NPs annealed at *T*_A_ = 473 K were not coarsening and maintained nanometer size with low thermal conductivity. Spin Seebeck voltages clearly appeared in the [Ta and Pt film/Fe_3_O_4_ NP]_1_ multilayered films annealed at *T*_A_ = 473 K under temperature differences. We found that the thermoelectric power of [Pt film/Fe_3_O_4_ NP]_*n*_ films on a polyimide sheet increases with increasing *n* while maintaining sufficient flexibility. The spin Seebeck effect voltage of *N*-Pt thermopile devices, which consists of *N* [Pt film/Fe_3_O_4_ NP]_1_ pieces and *N*−1 [Ta film/Fe_3_O_4_ NP]_1_ pieces, is found sufficiently larger than that of single [Pt film/Fe_3_O_4_ NP]_1_ piece. Finally, a 3-Pt thermopile device using the cut [Ta and Pt film/Fe_3_O_4_ NP]_5_ pieces was fabricated. In this device, not only a high thermoelectric voltage was obtained by serial circuit but also high power was realized in the multilayers. These results indicate that the spin Seebeck thermoelectric generation using easy fabrication process could be enhanced without the need for lithography techniques. Therefore, Fe_3_O_4_ NP-assembled films show high potential for both high scalability and sufficient flexibility.

## Data Availability

The data that support the findings of this study are available from the corresponding author upon reasonable request.
